# Development of functional gastrointestinal disorders after *Giardia lamblia *infection

**DOI:** 10.1186/1471-230X-9-27

**Published:** 2009-04-21

**Authors:** Kurt Hanevik, Vernesa Dizdar, Nina Langeland, Trygve Hausken

**Affiliations:** 1 Institute of Medicine, University of Bergen, 5021 Bergen, Norway; 2 Department of Medicine, Haukeland University Hospital, 5021 Bergen, Norway

## Abstract

**Background:**

Functional gastrointestinal disorders (FGID) may occur following acute gastroenteritis. This long-term complication has previously not been described after infection with the non-invasive protozoan *Giardia lamblia*. This study aims to characterize persistent abdominal symptoms elicited by *Giardia *infection according to Rome II criteria and symptoms scores.

**Methods:**

Structured interview and questionnaires 12–30 months after the onset of *Giardia *infection, and at least 6 months after *Giardia *eradication, among 82 patients with persisting abdominal symptoms elicited by the *Giardia *infection. All had been evaluated to exclude other causes.

**Results:**

We found that 66 (80.5%) of the 82 patients had symptoms consistent with irritable bowel syndrome (IBS) and 17 (24.3%) patients had functional dyspepsia (FD) according to Rome II criteria. IBS was sub classified into D-IBS (47.0%), A-IBS (45.5%) and C-IBS (7.6%). Bloating, diarrhoea and abdominal pain were reported to be most severe. Symptoms exacerbation related to specific foods were reported by 45 (57.7%) patients and to physical or mental stress by 34 (44.7%) patients.

**Conclusion:**

In the presence of an IBS-subtype pattern consistent with post-infectious IBS (PI-IBS), and in the absence of any other plausible causes, we conclude that acute *Giardia *infection may elicit functional gastrointestinal diseases with food and stress related symptoms similar to FGID patients in general.

## Background

Long term abdominal symptoms may develop after acute gastroenteritis and was first described in 1962 [1]. These symptoms are clinically similar to functional gastrointestinal diseases (FGID) and may be classified using the Rome II criteria for such illnesses. Symptoms often fulfil the criteria for irritable bowel syndrome (IBS) and the term post-infectious irritable bowel syndrome (PI-IBS) is often used for this condition [2]. It has been shown to occur following viral, bacterial and amoebic gastroenteritis and after trichinellosis [1,3,4]. A meta-analysis found the odds of developing irritable bowel syndrome (IBS) to be increased sixfold after acute gastroenteritis [5]. Previously, *Giardia *infection has been found to trigger abdominal symptoms in patients with established IBS[6], and *Giardia *should be ruled out as a possible cause in patients with IBS-like symptoms[7]. However, post-infectious functional gastrointestinal diseases elicited by infection with the non-invasive protozoan *Giardia lamblia *have not been described before. Similarly, the relation of patients' abdominal symptoms and food types and the influence of physical or mental stress have been well researched among IBS-patients in general[8,9], but little data exists regarding such relations in post-infectious FGIDs.

*Giardia lamblia *is a parasite of the small intestine occurring endemically, or as the cause of waterborne outbreaks. The parasite is commonly found in children in developing countries and in travellers to endemic regions. It causes infections varying from asymptomatic to protracted and severe illness with diarrhoea, weight loss and malabsorbtion[10]. After an outbreak in autumn 2004 of assemblage B giardiasis in Bergen, Norway, many patients experienced long-lasting abdominal symptoms despite one or several courses of metronidazole. They were referred to the local university hospital for evaluation. Extensive work-up revealed a surprisingly high rate of duodenal inflammation both in patients with and without evidence of chronic infection [11]. All *Giardia *positive patients were treated successfully[12]. However, symptoms remained in many patients despite eradication of the parasite, and they were followed up regularly.

The main aim of this study was to evaluate the abdominal symptoms according to the Rome II criteria for FGID among patients with persisting abdominal symptoms 12–30 months after the onset of *Giardia *infection, and more than 6 months after *Giardia *eradication. Secondarily, we included some questions about the symptoms relation to food types and stress.

## Methods

This is a prospective study describing the results of questionnaires and structured interviews during follow-up visits of patients with persisting symptoms following *Giardia *infection. The data were collected during a 16 month period from January 2006 until April 2007.

All 82 patients included in this study had laboratory confirmed giardiasis during an outbreak in 2004. They had been successfully treated and were confirmed *Giardia *negative by microscopy of three or more faecal samples at least 6 months prior to inclusion in this study. An extensive work-up including upper endoscopy with duodenal biopsies, routine blood screening tests, immunoglobulins, IgE, serum anti-endomysial, anti-tissue transglutaminase antibodies and faecal calprotectin had not produced any findings to explain their symptoms [11]. In 11 patients included in this study, repeated routine duodenal biopsies were taken approximately one year after the initial work-up, and these were reviewed with regard to inflammatory changes.

After the initial extensive work-up, patients came for follow up visits where, in addition to routine clinical examination, a structured interview was performed with regard to previous abdominal symptoms, and symptom exacerbation related to food types or physical or mental stress. In the interview most patients denied previous abdominal illness, while 14 (17.1%) had experienced some previous abdominal symptoms. All these 14 patients reported considerable more abdominal problems after *Giardia *infection than they had had before. Only 5 of them reported previous contact with a physician about their abdominal problems. For clarity we chose to give results for patients without previous abdominal complaints separately in the analysis.

Patients also filled in a questionnaire regarding their current abdominal symptoms allowing evaluation of IBS according to the Rome II criteria[13]. As it became evident that functional dyspepsia (FD) was occurring in our patient population, Rome II FD criteria questions were included in the questionnaires. IBS patients were sub classified into diarrhoea-predominant IBS (D-IBS: two or more diarrhoea symptoms and a maximum of one symptom of constipation), constipation-predominant IBS (C-IBS: two or more constipation symptoms and a maximum of one symptom of diarrhoea) and alternating IBS (A-IBS: all subjects with IBS not qualifying for D-IBS or C-IBS). Symptom severity, for the last month, of nausea, bloating, abdominal pain, diarrhoea, constipation and anorexia was quantified by patients grading these symptoms on an ordinal scale from 0 – 10 with 0 = no symptoms and 10 = severe symptoms.

Statistical analysis was performed using SPSS 16.0. The Regional ethics committee and the Norwegian Social Science Data Services approved the data collection and analysis of the data.

## Results

There were 52 females (63.4%) and the mean age was 31.8 years (range 18–61). Analysis of the collected data showed a high frequency of patient's symptoms fulfilling the Rome II IBS criteria with D-IBS and A-IBS of similar prevalence (Table 1). IBS-C was uncommon. Regarding FD, 17 cases were identified and 10 of these also had concurrent IBS. The 6 cases fulfilling neither FD nor IBS criteria could be put into one or more of the Rome II categories; functional abdominal pain, functional diarrhoea and functional bloating.

**Table 1:** Frequency of FGID and relation of abdominal symptoms to food types and stress

	Patients **without **previous abdominal symptoms N = 68		All patients N = 82	
	*N*	*%*	*N*	*%*
IBS all subtypes	54	79.4	66	80.5
D – IBS^1^	25	46.3	31	47.0
A – IBS^1^	27	50.0	30	45.5
C – IBS^1^	3	5.6	5	7.6
FD^2^	14	21.9	17	24.3
IBS & FD^2^	8	12.5	10	14.3
Other FGID (not IBS/FD)	6	8.8	6	7.3
Food related symptoms^3^	37	56.1	45	57.7
Stress related symptoms^4^	26	40.6	34	44.7

^1 ^Percentages within subgroup with IBS

^2 ^70 patients with available FD data

^3 ^78 patients with available food related data

^4 ^76 patients with available stress related data

No significant sex differences were found regarding IBS subtypes, symptom scores, influence of stress or subjective food intolerance, only trends towards more FD among females (p = 0.09) and more females reporting previous abdominal symptoms (p = 0.07). Patients with FD had similar symptom scores as non-FD patients except for the constipation score which was 2.85 in FD patients and 0.65 in non-FD patients with a p-value < 0.001.

Bloating was the symptom reported to be most severe (Table 2). In fact only two patients reported nil bloating. Symptoms of diarrhoea and constipation varied consistently with IBS subtype. No gender differences were found, only a trend towards nausea being more severe among females (p = 0.08).

**Table 2:** Symptom scores in IBS subtypes and in the group with other FGIDs.

Symptom	D-IBS N = 31	A-IBS N = 30	C-IBS N = 5	Other FGID N = 16	All N = 82
Nausea	2.8 ± 0.4	3.1 ± 0.5	3.4 ± 1.5	3.0 ± 0.8	3.0 ± 0.3
Bloating	6.2 ± 0.4	7.0 ± 0.4	8.0 ± 0.8	5.4 ± 0.8	6.4 ± 0.2
Abd.pain	3.9 ± 0.5	4.6 ± 0.4	5.2 ± 1.0	3.8 ± 0.6	4.2 ± 0.3
Constipation	1.7 ± 0.5	3.1 ± 0.5	3.8 ± 1.2	0.9 ± 0.3	2.2 ± 0.3
Diarrhea	5.4 ± 0.4	4.5 ± 0.5	3.4 ± 1.0	4.3 ± 0.7	4.8 ± 0.3
Anorexia	2.0 ± 0.5	1.5 ± 0.4	2.4 ± 0.8	1.1 ± 0.5	1.7 ± 0.3

Total score	22.1 ± 1.5	23.9 ± 1.6	26.2 ± 5.0	19.1 ± 2.1	22.3 ± 1.0

Values given are mean scores ± standard error.

The question of food related worsening of symptoms was answered by 78 patients. The majority of patients (57.7%) reported their post-giardiasis abdominal problems to worsen after intake of certain food items. They were asked to name the food items they had begun to avoid. Milk and milk products were mentioned spontaneously by 27% of the patients. Other common food items mentioned were alcohol containing beverages (18.4%), wheat flour products (14.5%) and coffee (6%).

In 76 patients who responded to the question about the influence of physical or mental stress on their abdominal symptoms, 44.7% felt that their abdominal illness was worsened by stress. No correlations were found between IBS-subtypes and symptoms exacerbation related to stress and food types.

Signs of inflammation with infiltration of inflammatory cells, with or without shortening and blunting of intestinal villi, were found in 8 out of 11 of these patients during the first workup in spring 2005. One year later, repeated routine duodenal biopsies were normal in ten and improved in one of these patients, although all these patients still had troubling abdominal symptoms (data not shown).

## Discussion

Clinical characteristics of a relatively large number of patients seeking help for long lasting abdominal symptoms after *Giardia *infection are described in this study. An extensive follow-up of these patients over three years has not revealed any specific illness, which can explain the symptoms seen in our study population. The prolonged symptoms in parasitologically successfully treated patients came as a surprise, as such complications have not been described after *Giardia *infection before.

There have been several *Giardia *outbreaks described, among others in Solna, Sweden[14], Creston, Canada [15] and Aspen highlands, USA[16]. However, no follow-up studies have looked at persistent symptoms after eradication of the parasite. An epidemiological study in Michigan, USA[17] did not find any link between *Giardia *infection and IBS, by correlating new *Giardia *cases with prescriptions of three drugs (dicyclomine, tegaserod and alosetron) used in IBS. In our study population any such correlation would probably also not have been found as patients were previously largely healthy, active young people unaccustomed to taking drugs daily. Only a few had abdominal complaints of a severity that any of these medications would be considered.

In our study population we find a pattern of IBS-subtypes with a high frequency of diarrhoeal symptoms and little constipation. This agrees well with previous descriptions of PI-IBS as a distinctive subgroup of IBS patients [18]. In the general population in Norway a recent study with 4622 respondents showed that 10% in the relevant age group fulfilled the IBS criteria and the pattern of subtypes contrasts our findings with subtype A-IBS most commonly reported (53%), followed by similar prevalence of the other two subtypes D-IBS (23%) and C-IBS (24%) [19].

It is known that *Giardia *may cause prolonged symptoms for several weeks after successful treatment due to secondary lactose intolerance [20]. In the present study we found that many patients reported many different kinds of food to worsen symptoms and that this persisted for years after infection. A previous study has not found lactose-intolerance to be factor in the aetiology of PI-IBS after bacterial gastroenteritis[21]. Preliminary data from duodenal lactase activity testing in newly referred patients with post-giardiasis IBS at our hospital support this finding (unpublished).

It is known that food-related gastrointestinal symptoms are common in the general population and often coincides with IBS. A previous study found that 51% of IBS-patient considered that their symptoms were linked to individual foods [8], and improvement following exclusion diets have also been reported [22]. However, objective measurements methods, like skin prick test or intestinal permeability have not been consistent with the reported food-intolerance [23]. Outside of this study many of the patients were referred to allergologic evaluation with no specific findings. It thus seems that the food-related symptoms seen in our patient population may be intrinsically linked with the development of FGID.

The finding that around half the patients felt that physical or mental stress influence their abdominal symptoms is consistent with previous findings in patients with IBS of all causes seeking medical care in Norway [9].

It remains speculative what may be the reason for development of FGID after *Giardia *infection. Previous studies have pointed to psychological factors, young age, and severity and duration of the acute infection as factors increasing the risk of FGID after bacterial gastroenteritis [2,5]. We showed in a former study that a high frequency of microscopic duodenal inflammation was found in our study population when illness duration was 2–4 months, indicating that the severity of host response may be a risk factor for FGID in our population [11]. The initially high frequency of duodenal microscopic inflammation normalised within a year, but subtle low-grade inflammation, not recognised by routine microscopy might be ongoing.

The particular genotype of *Giardia *responsible for the epidemic may also be a relevant risk factor in itself, as different strains have been shown to differ in their ability to induce small intestinal injury in rats [24]. The higher proportion of females in our study population is probably not indicating an increased risk, but rather a reflection of more females contracting giardiasis during the epidemic, probably as a consequence of higher water intake [25]. Host factors like age, no previous *Giardia *exposure or infection, pre-existing intestinal microbiota, immunologic and genetic predisposition may also play a role. These will be interesting issues for further research.

All patients were treated with one or more courses of metronidazole for their giardiasis. Although one study points to a link between antibiotic treatment in general and FGID[26], prolonged gastrointestinal symptoms is not reported as a side effect of metronidazole. Although we can not fully exclude this possibility, we think the *Giardia *infection is a far more plausible cause of the later FGID than its treatment.

A strength of this study is the relatively large number of laboratory confirmed giardiasis cases who have been thoroughly investigated and followed for a long period of time. However, it is therefore also important to note that our data are drawn from a population likely to be the more severely affected, and may not represent patients with milder post-giardiasis abdominal symptoms. A control group presented with the same follow-up and questionnaire would have been desirable.

Patients were asked about previous abdominal complaints retrospectively, one to two years after their *Giardia *infection. Recall bias might influence the answers given. Some caution should also be taken regarding the exclusion of chronic giardiasis. Microscopy of three stool samples for *Giardia *cysts has a sensitivity of 85 – 90%[27]. Our patient population had repeated series of *Giardia *negative faecal samples before referral and during the hospital workup, thus we consider the risk of low-grade chronic infections to be very small.

## Conclusion

Due to the subtype pattern consistent with PI-IBS, no other discernable cause, and symptoms being elicited by a symptomatic, laboratory confirmed *Giardia *infection, we here document for the first time that the non-invasive protozoan pathogen *Giardia lamblia *may induce irritable bowel syndrome and functional dyspepsia. Utilising the unique setting of around 1300 laboratory confirmed *Giardia *cases, we are planning follow up studies of the frequency and characteristics of FGID after *Giardia *infection including population based approaches.

## Competing interests

The authors declare that they have no competing interests.

## Authors' contributions

Data collection was done by KH and VD. TH and NL supervised the study. All participated in the writing and finalisation of the paper. All authors have approved the final draft submitted.

## Pre-publication history

The pre-publication history for this paper can be accessed here:

http://www.biomedcentral.com/1471-230X/9/27/prepub

## References

[B1] Chaudhary NA Truelove SC The irritable colon syndrome. A study of the clinical features, predisposing causes, and prognosis in 130 cases Q J Med 1962 31 307 322 13878459

[B2] Spiller RC Is IBS caused by infectious diarrhea? Nat Clin Pract Gastroenterol Hepatol 2007 4 642 643 10.1038/ncpgasthep0983 17984983

[B3] Marshall JK Thabane M Garg AX Clark WF Salvadori M Collins SM Incidence and epidemiology of irritable bowel syndrome after a large waterborne outbreak of bacterial dysentery Gastroenterology 2006 131 445 450 10.1053/j.gastro.2006.05.053 16890598

[B4] Soyturk M Akpinar H Gurler O Pozio E Sari I Akar S Irritable bowel syndrome in persons who acquired trichinellosis Am J Gastroenterol 2007 102 1064 1069 10.1111/j.1572-0241.2007.01084.x 17313500

[B5] Thabane M Kottachchi DT Marshall JK Systematic review and meta-analysis: The incidence and prognosis of post-infectious irritable bowel syndrome Aliment Pharmacol Ther 2007 26 535 544 17661757 10.1111/j.1365-2036.2007.03399.x

[B6] D'Anchino M Orlando D De Feudis L Giardia lamblia infections become clinically evident by eliciting symptoms of irritable bowel syndrome J Infect 2002 45 169 172 10.1016/S0163-4453(02)91038-8 12387773

[B7] Grazioli B Matera G Laratta C Schipani G Guarnieri G Spiniello E Giardia lamblia infection in patients with irritable bowel syndrome and dyspepsia: a prospective study World J Gastroenterol 2006 12 1941 1944 16610003 10.3748/wjg.v12.i12.1941 PMC4087522

[B8] Simren M Mansson A Langkilde AM Svedlund J Abrahamsson H Bengtsson U Food-related gastrointestinal symptoms in the irritable bowel syndrome Digestion 2001 63 108 115 11244249 10.1159/000051878

[B9] Vandvik PO Wilhelmsen I Ihlebaek C Farup PG Comorbidity of irritable bowel syndrome in general practice: a striking feature with clinical implications Aliment Pharmacol Ther 2004 20 1195 1203 10.1111/j.1365-2036.2004.02250.x 15569123

[B10] Ortega YR Adam RD Giardia: Overview and update Clinical Infectious Diseases 1997 25 545 549 10.1086/513745 9314441

[B11] Hanevik K Hausken T Morken MH Strand EA Morch K Coll P Persisting symptoms and duodenal inflammation related to Giardia duodenalis infection J Infect 2007 55 524 530 10.1016/j.jinf.2007.09.004 17964658

[B12] Morch K Hanevik K Robertson LJ Strand EA Langeland N Treatment-ladder and genetic characterisation of parasites in refractory giardiasis after an outbreak in Norway J Infect 2008 56 268 273 10.1016/j.jinf.2008.01.013 18328567

[B13] Thompson WG Longstreth GF Drossman DA Heaton KW Irvine EJ Muller-Lissner SA Functional bowel disorders and functional abdominal pain Gut 1999 45 Suppl 2 II43 II47 10457044 10.1136/gut.45.2008.ii43 PMC1766683

[B14] Ljungstrom I Castor B Immune response to Giardia lamblia in a water-borne outbreak of giardiasis in Sweden J Med Microbiol 1992 36 347 352 1588586 10.1099/00222615-36-5-347

[B15] Isaac-Renton JL Cordeiro C Sarafis K Shahriari H Characterization of Giardia duodenalis isolates from a waterborne outbreak J Infect Dis 1993 167 431 440 8421176 10.1093/infdis/167.2.431

[B16] Istre GR Dunlop TS Gaspard GB Hopkins RS Waterborne giardiasis at a mountain resort: evidence for acquired immunity Am J Public Health 1984 74 602 604 6721017 10.2105/AJPH.74.6.602 PMC1651646

[B17] Penrose AS Wells EV Aiello AE Infectious causation of chronic disease: examining the relationship between Giardia lamblia infection and irritable bowel syndrome World J Gastroenterol 2007 13 4574 4578 17729408 10.3748/wjg.v13.i34.4574 PMC4611829

[B18] Dunlop SP Jenkins D Spiller RC Distinctive clinical, psychological, and histological features of postinfective irritable bowel syndrome Am J Gastroenterol 2003 98 1578 1583 10.1111/j.1572-0241.2003.07542.x 12873581

[B19] Vandvik PO Lydersen S Farup PG Prevalence, comorbidity and impact of irritable bowel syndrome in Norway Scand J Gastroenterol 2006 41 650 656 10.1080/00365520500442542 16716962

[B20] Farthing MJ Giardiasis Gastroenterol Clin North Am 1996 25 493 515 10.1016/S0889-8553(05)70260-0 8863037

[B21] Parry SD Barton JR Welfare MR Is lactose intolerance implicated in the development of post-infectious irritable bowel syndrome or functional diarrhoea in previously asymptomatic people? Eur J Gastroenterol Hepatol 2002 14 1225 1230 10.1097/00042737-200211000-00010 12439117

[B22] Nanda R James R Smith H Dudley CR Jewell DP Food intolerance and the irritable bowel syndrome Gut 1989 30 1099 1104 2767507 10.1136/gut.30.8.1099 PMC1434180

[B23] Dainese R Galliani EA De Lazzari F Di LV Naccarato R Discrepancies between reported food intolerance and sensitization test findings in irritable bowel syndrome patients Am J Gastroenterol 1999 94 1892 1897 10.1111/j.1572-0241.1999.01226.x 10406255

[B24] Cevallos A Carnaby S James M Farthing JG Small intestinal injury in a neonatal rat model of giardiasis is strain dependent Gastroenterology 1995 109 766 773 10.1016/0016-5085(95)90383-6 7657104

[B25] Nygard K Schimmer B Sobstad O Walde AK Tveit I Langeland N A large community outbreak of waterborne giardiasis-delayed detection in a non-endemic urban area BMC Public Health 2006 6 141 16725025 10.1186/1471-2458-6-141 PMC1524744

[B26] Maxwell PR Rink E Kumar D Mendall MA Antibiotics increase functional abdominal symptoms Am J Gastroenterol 2002 97 104 108 10.1111/j.1572-0241.2002.05428.x 11808932

[B27] Goka AK Rolston DD Mathan VI Farthing MJ The relative merits of faecal and duodenal juice microscopy in the diagnosis of giardiasis Trans R Soc Trop Med Hyg 1990 84 66 67 10.1016/0035-9203(90)90386-S 2345928

